# 769 Racial, Ethnic, and Socioeconomic Disparities in Burn Care Access

**DOI:** 10.1093/jbcr/irad045.244

**Published:** 2023-05-15

**Authors:** Julia Tomtschik, Keith Sweitzer, Caitlin Cook, Aidan O'Shea, Derek Bell

**Affiliations:** University of Rochester Medical Center, Rochester, New York; University of Rochester Medical Center, Rochester, New York; University of Rochester Medical Center, Rochester, New York; University of Rochester Medical Center, Rochester, New York; University of Rochester Medical Center, Rochester, New York; University of Rochester Medical Center, Rochester, New York; University of Rochester Medical Center, Rochester, New York; University of Rochester Medical Center, Rochester, New York; University of Rochester Medical Center, Rochester, New York; University of Rochester Medical Center, Rochester, New York; University of Rochester Medical Center, Rochester, New York; University of Rochester Medical Center, Rochester, New York; University of Rochester Medical Center, Rochester, New York; University of Rochester Medical Center, Rochester, New York; University of Rochester Medical Center, Rochester, New York; University of Rochester Medical Center, Rochester, New York; University of Rochester Medical Center, Rochester, New York; University of Rochester Medical Center, Rochester, New York; University of Rochester Medical Center, Rochester, New York; University of Rochester Medical Center, Rochester, New York; University of Rochester Medical Center, Rochester, New York; University of Rochester Medical Center, Rochester, New York; University of Rochester Medical Center, Rochester, New York; University of Rochester Medical Center, Rochester, New York; University of Rochester Medical Center, Rochester, New York

## Abstract

**Introduction:**

While racial, ethnic, and socioeconomic disparities in burn care have been identified, there is a paucity of research into specific means by which these disparities manifest. Here, we consider time to initial burn surgery consult.

**Methods:**

Through a retrospective review of electronic health records, we reviewed all patients evaluated by the burn surgery service at a single regional burn center from Jun 2020 to Apr 2022. We excluded patients without data for time of onset of burn injury. We analyzed differences in time to burn consult, rate of surgical management, and average length of stay (LOS), stratifying by patient race (Caucasian, Black, Asian, Native American, Other), ethnicity (Latino, non-Latino, Other), and insurance payor (Medicaid, Private, Medicare, Other, None). Time to burn consult was defined as time from onset of burn injury to time of burn consult.

**Results:**

365 patients met the inclusion criteria. Average age was 33.3 years; 65.8% were male. Average (standard deviation) time to burn consult was 17h7m (1d23h21m). No significant differences in time to burn consult existed when the study group was stratified by race, ethnicity, or insurance payor. Rate of surgical management (Chi-squared p=0.05) and LOS (ANOVA p< 0.0001) differed by insurance payor, but not race or ethnicity. Medicare patients had higher rates of surgical intervention and longer average LOS; patients with no insurance had lower rates of surgical intervention and shorter average LOS.

**Conclusions:**

Our results indicate that time to burn consult is unlikely to contribute meaningfully to racial, ethnic, and socioeconomic disparities in burn care. Further studies are needed to better understand other aspects of care that may contribute to these disparities.

**Applicability of Research to Practice:**

Time to burn consult is unlikely to contribute to disparities in burn care. Burn care providers should be mindful of other ways that disparities may manifest.

